# Clinical Implication of Individually Tailored Segmentation Method for Distorted Hypothalamus in Craniopharyngioma

**DOI:** 10.3389/fendo.2021.763523

**Published:** 2021-12-20

**Authors:** A Ram Hong, Miwoo Lee, Jung Hyun Lee, Jung Hee Kim, Yong Hwy Kim, Hyung Jin Choi

**Affiliations:** ^1^ Department of Internal Medicine, Chonnam National University Medical School, Gwangju, South Korea; ^2^ Department of Anatomy, Seoul National University College of Medicine, Seoul, South Korea; ^3^ Department of Pituitary Center, Seoul National University College of Medicine, Seoul, South Korea; ^4^ Department of Neurosurgery, Seoul National University College of Medicine, Seoul, South Korea; ^5^ Department of Internal Medicine, Seoul National University College of Medicine, Seoul, South Korea

**Keywords:** craniopharyngioma, hypothalamic obesity, hypothalamus volume, magnetic resonance imaging, surgery

## Abstract

**Objective:**

Several attempts have been done to capture damaged hypothalamus (HT) using volumetric measurements to predict the development of hypothalamic obesity in patients with craniopharyngioma (CP). This study was to develop a novel method of HT volume measurement and examine the associations between postoperative HT volume and clinical parameters in patients with CP.

**Methods:**

We included 78 patients with adult-onset CP who underwent surgical resection. Postoperative HT volume was measured using T1- and T2-weighted magnetic resonance imaging (MRI) with a slice thickness of 3 mm, and corrected for temporal lobe volume. We collected data on pre- and postoperative body weights, which were measured at the time of HT volume measurements.

**Results:**

The corrected postoperative HT volume measured using T1- and T2-weighted images was significantly correlated (*r*=0.51 [95% confidence interval (CI) 0.32 to 0.67], *P*<0.01). However, HT volume was overestimated using T1-weighted images owing to obscured MR signal of the thalamus in patients with severe HT damage. Therefore, we used T2-weighted images to evaluate its clinical implications in 72 patients with available medical data. Postoperative HT volume was negatively associated with preoperative body weight and preoperative tumor volume (*r*=–0.25 [95% CI -0.45 to -0.04], *P*=0.04 and *r*=–0.26 [95% CI -0.40 to -0.15], *P*=0.03, respectively). In the subgroup analysis of CP patients who underwent primary surgery (*n*=56), pre- and postoperative body weights were negatively associated with HT volume (*r*=–0.30 [95% CI -0.53 to -0.03], *P*=0.03 and *r*=–0.29 [95% CI -0.53 to -0.02], *P*=0.03, respectively).

**Conclusions:**

Adult-onset CP patients showed negative associations between postoperative HT volume and preoperative/postoperative body weight using a new method of HT volume measurement based on T2-weighted images.

## Introduction

Patients with craniopharyngioma (CP) exhibit excessive weight gain, which is commonly referred to as hypothalamic obesity (HO) (1). HO develops in 30–70% of patients with CP after surgery and/or radiotherapy, which leads to increased negative metabolic consequences and mortality (2, 3). Hypothalamic injury, particularly in the regions that regulate appetite and balance energy metabolism, is considered to play a major role in the development of postoperative weight gain in patients with CP (4). Patients with HO commonly exhibit hyperphagia, reduced energy expenditure, low resting metabolic rate, and low physical activity (5–7). Dysregulation of circadian rhythm and reduced sympathetic tone are also suggested as possible factors for the development of HO (8, 9).

Based on the pathophysiological mechanisms associated with HO, the occurrence and degree of this condition inevitably depend on the damaged hypothalamic region. The ventromedial hypothalamus (HT) is the most crucial territory for the development of HO and includes two major nuclei, the arcuate nucleus and paraventricular nucleus (7). The arcuate nucleus regulates appetite, in which agouti-related protein and neuropeptide Y increases appetite, whereas proopiomelanocortin neurons decrease appetite (10). Thus, hypothalamic injury, particularly involving the ventromedial hypothalamus, can lead to dysregulation of appetite, which contributes to the development of HO. Disruption of the posterior hypothalamic nuclei, including the dorsomedial nucleus, ventromedial nucleus, and dorsal hypothalamic area nucleus, has been suggested as a crucial factor for the occurrence of HO. Rather than regulating appetite, these nuclei mainly mediate leptin-induced energy expenditure and sympathetic activation related to locomotion and thermogenesis (11).

To date, several studies have attempted to analyze hypothalamic damage in patients with CP to predict HO. Roth et al. developed a novel hypothalamic lesion scoring system that covers hypothalamic areas critical to energy homeostasis (12). This approach scores the affected lesions using anatomical landmarks in both sagittal and coronal views, not measuring HT volume. The study showed that although anterior and medial hypothalamic injuries were commonly observed in patients with HO, the most robust weight gain was observed in patients with disruptions in the posterior hypothalamus. However, the sample size of the study was small (41 patients with CP) and all patients had childhood-onset CP. Fjalldal et al. used a manual segmentation method to measure HT volume involving 3T magnetic resonance imaging (MRI) and showed negative associations of HT volume with fat mass and leptin (13). However, this study also included only patients with childhood-onset CP, with a median age of 22 years after the initial diagnosis.

In the current study, we aimed to evaluate hypothalamic damage in adult patients with CP using HT volume measurements. To better evaluate hypothalamic damage, we first developed a method for HT volume measurements using T2-weighted MR images and compared its performance with that of T1-weighted MR images. Based on HT volume data using T2-weighted MR images, we further analyzed the associations between the postoperative HT volume and clinical parameters in adult patients with CP.

## Methods

### Development of Individually Tailored HT Volumetric Method

We included 78 (42 male and 36 female) adult-onset CP patients (aged ≥ 18 years) who underwent surgical resection at Seoul National University Hospital (SNUH) between 2012 and 2017. Two independent raters manually segmented the HT area using the freehand option of the SNUH PACS program (INFINITT Co. Ltd, South Korea). Rater 1 (M.L.) was a well-trained neuroimaging analyst. Rater 2 (Y.H.K.) was an expert neurosurgeon who conducted all surgical resections.

#### Method 1: Conventional Method (*n*=62)

**Figure 1:**
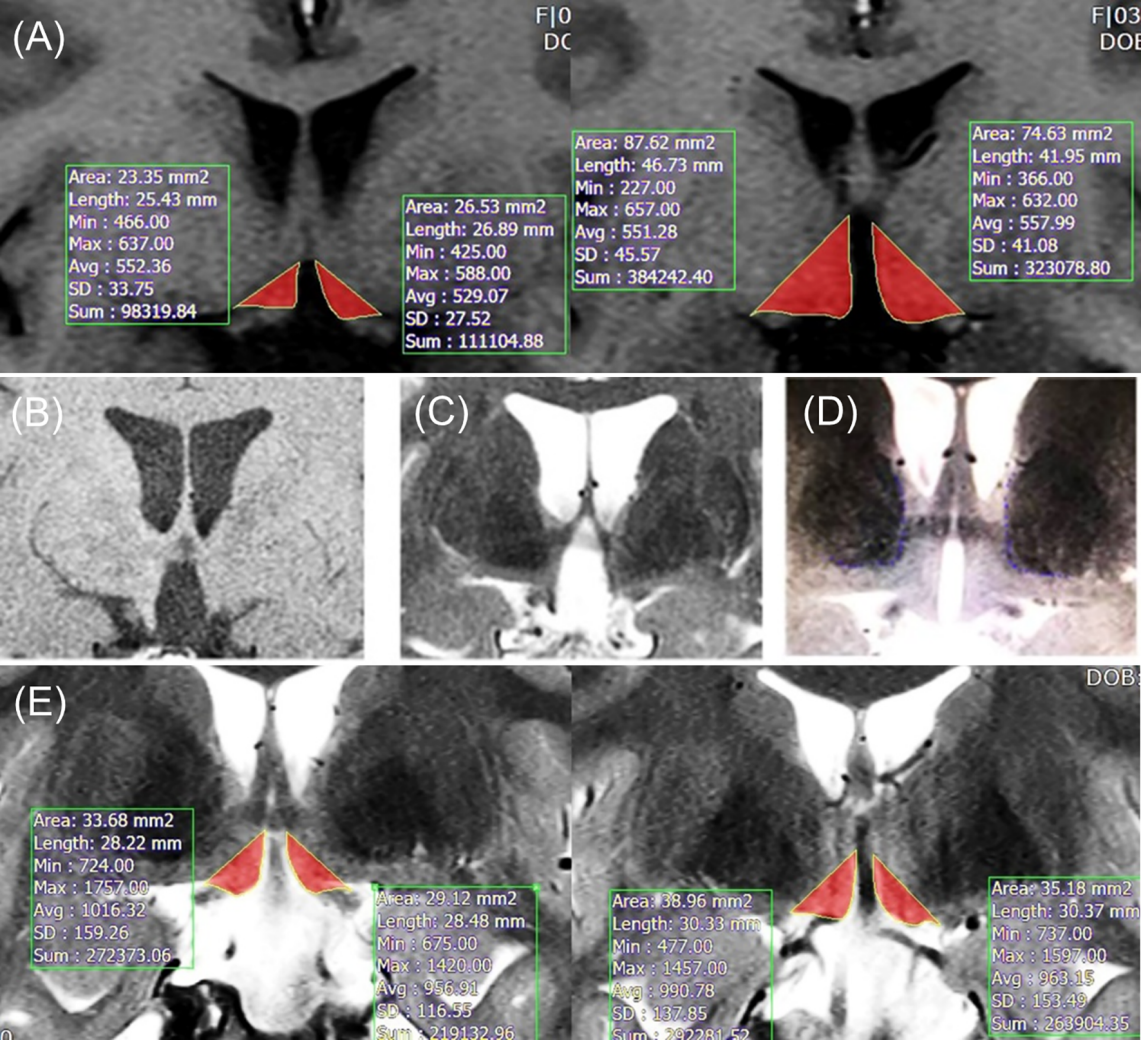
**(A)** Example of method 1 in T1-weighted images. **(B)** T1-weighted image with severely damaged HT (optic tract displacement is not observed). **(C)** T2-weighted image with severely damaged HT (optic tract displacement is observed). **(D)** T2-weighted image with normal HT. **(E)** Example of individually tailored method in T2-weighted images. Lateral borderline (the edge of optic tract) was adjusted for each patient’s thalamus borderline. HT, hypothalamus.

Rater 1 evaluated the HT volume in 62 subjects. Two to five slices (3 mm thickness) were taken for HT volumetric analysis, from the slice where optic tract splits from optic chiasm to just before the slice where the mammillary body is visible, from postoperative T1-weighted MR images (Discovery 750w 3.0 Tesla scanner, General Eletronics Company) at the last follow-up. T1-weighted MR images were obtained using the following parameters: Slice number=15, TR=416.7 ms, echo time=11 ms, flip angle=90, voxel size=0.4 x 0.5 x 3 mm^3^, slice thickness=3 mm, and matrix=320 x 256. Rater 1 manually segmented the HT area by established boundaries of HT: the lateral edge of the optic tract, third ventricle, optic chiasm, and mammillary body (13, 14). Lateral border of hypothalamus defined as a linear line was drawn connecting upper edge of third ventricle and lateral edge of optic tract. The raw HT volume (left and right separately, mm³) of each subject “i” was calculated as follows: Average segmented area of slices (mm²) x slice number of “i” x inter-slice space (slice thickness, mm). **Figure 1A** shows an example of HT volume measurements using T1-weighted MR images.

#### Method 2: T2-Based Individually Tailored Method (*n*=78)

Method 2 was separately operated by rater 1 and 2. In patients with severely damaged HT, the optic tract is displaced outwardly owing to the large space of the third ventricle. In this situation, the HT volume can be distorted by the displacement of the optic tract location, which is detected on a T2-weighted image, but not on a T1-weighted image (**Figures 1B–D**). To adjust the bias resulting from this, we adopted a new segmentation method using T2-weighted images instead of T1-weighted images. T2-weighted images were scanned using the following parameters: Slice number=15, voxel size=0.4 x 0.5 x 3 mm^3^, matrix=320 x 256, slice thickness=3 mm, TR=3000 ms, eco time=127.2 ms, and flip angle=160. Medial ventral border of thalamus was visualized using T2 image. For patients with minimal lateral distortion, who did not have any overlap between the lateral border of hypothalamus and medial ventral border of thalamus, conventional method was used to measure HT volume. However, for patient with more severe lateral distortion, there was substantial overlap between the lateral border of hypothalamus and medial ventral border of thalamus. For these patients with more severe lateral distortion, lateral border of hypothalamus was defined as a linear line from third ventricle upper edge to medial ventral border of thalamus (leading to more medial position than lateral edge of optic tract). The lateral borderline was tailored to adjust the boundary of the thalamus for each patient. An example of an individually tailored method using T2-weighted images is shown in **Figure 1E**.

#### Whole Brain Volume Correction

Three-dimensional segmental volume measurement of the temporal lobe was performed to adjust for whole brain size and brain atrophy (images of whole brain were unavailable). We measured temporal lobe volume to minimize changes in brain parenchymal volume resulting from hydrocephalus or mass effect. Temporal lobe volume has been reported to be associated with whole brain volume (15–17). The temporal horn was excluded from the measurements. The temporal lobe volume was calculated separately for each side by multiplying the segmented area by the number of slices and the inter-slice space. The corrected HT volume of each subject ‘‘i’’ was computed as follows: (right or left) corrected HT volume (i) = (right or left) HT volume raw (i)/(right or left) temporal lobe volume (i), where ‘‘HT volume raw (i)’’ is the raw value of HT and ‘‘temporal lobe volume (i)’’ is the temporal lobe of the subject ‘‘i’’.

### Correlation Analysis Between Corrected Postoperative HT Volume and Clinical Parameters of Study Subjects

Among the 78 adult patients with CP, 6 patients were excluded from the analysis owing to lack of data on postoperative body weight or hormone replacement therapy. We collected pre- and postoperative height and body weight data measured at the time when MRI was performed for HT volume measurements. The time point at which the postoperative MRI was performed was individualized. Follow-up duration was determined by the interval between the pre- and postoperative weight measurements. We reviewed the medical records on the postoperative steroid hormone replacement and calculated the equivalent steroid dose of hydrocortisone to adjust for differences in the glucocorticoid potency between steroids. Pre- and postoperative subjective hypothalamic symptoms were assessed including sleeping disturbance, hyperphagia, and psychosocial problems. The presence of visual impairment was also examined. To assess hormone deficiency, basal hormone and dynamic function tests were performed in all patients before surgery and at 3 months postoperatively.

### Statistical Analysis

Data are expressed as means ± standard deviation or median (interquartile range) or n (%). Continuous variables were analyzed using Student’s t-test and categorical variables were analyzed using the chi-squared test. Pearson’s correlation coefficients and 95% confidence interval (CI) were calculated to assess the associations between the corrected postoperative HT volume and clinical parameters. All statistical analyses were performed using SPSS Statistics, version 25 (IBM, Armonk, NY, USA). *P*<0.05 was considered significant.

## Results

### Validation of the Individually Tailored HT Volumetric Method

**Figure 2:**
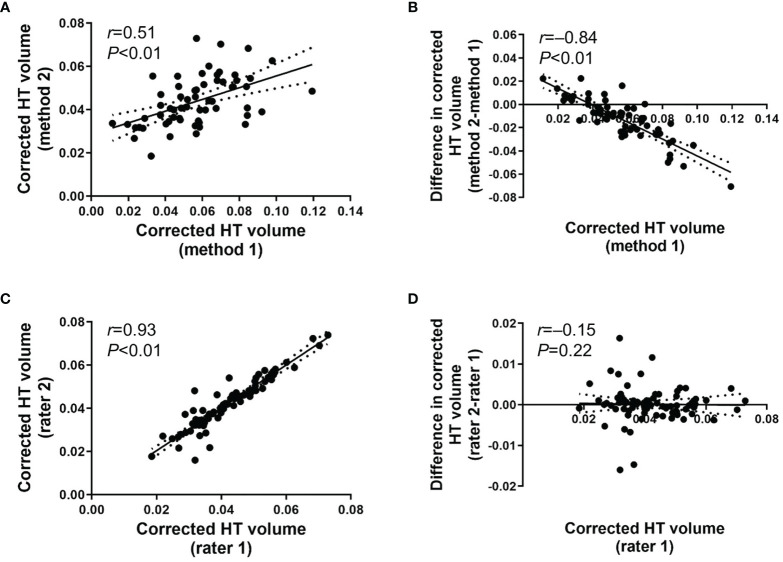
**(A)** Scatter plot for corrected HT volume between method 1 and method 2 operated by rater 1. **(B)** Bland-Altman plot for inter-method difference between method 1 and method 2 operated by rater 1. **(C)** Scatter plot for corrected HT volume using method 2 between rater 1 and rater 2. **(D)** Bland-Altman plot for inter-rater difference between rater 1 and rater 2 for method 2. Date are presented with 95% confidence interval.

We aimed to validate the performance and efficacy of the newly developed individually tailored HT volumetric method by comparing systematic bias and outliers between methods. Validation of the individually tailored HT volumetric method developed in the present study is shown in **Figure 2**. The corrected HT volume between method 1 and method 2 conducted by rater 1 was significantly correlated (*r*=0.51 [95% CI 0.32 to 0.67], *P*<0.01; **Figure 2A**), which suggests a good concordance and robustness between methods. The Bland–Altman plot revealed the relationship between the differences and the magnitude of the two measurement methods to identify any systematic bias and outliers. Systematic bias was observed between method 1 and method 2 of rater 1 (**Figure 2B**). The difference between method 1 and method 2 showed a significantly negative correlation with the magnitude of HT volume using method 1 (*r*=–0.84 [95% CI -0.91 to -0.72, *P*<0.01), which shows that there was a significant difference between methods in patients with smaller HT volumes. No significant outliers were observed. There was a significant correlation between the two raters’ HT volume assessment using method 2 (*r*=0.93 [95% CI 0.88 to 0.97], *P*<0.01; **Figure 2C**). Intraclass correlation (ICC) between the raters was reliable (ICC[2,1]=0.92 (95% CI 0.88 to 0.95), *P*<0.01). The Bland–Altman plot showed that there was a minimal systematic bias between raters 1 and 2 using method 2 (*r*=–0.15 [95% CI -0.32 to 0.12, *P*=0.22; **Figure 2D**). The variance of inter-rater difference was limited to an acceptable range (+0.02 to –0.02). Significant outliers were not observed. The individually tailored method (method 2) was superior to method 1 with respect to systematic bias, especially in cases of severe hypothalamic damage.

### Clinical Characteristics of Study Subjects

Baseline characteristics of the study subjects included in the correlation analysis are shown in **Table 1** (*n*=72). The mean age was 46.8 ± 14.8 years, and male patients were dominant (56.9%). The mean pre- and postoperative body weights were 69.1 ± 13.0 kg and 72.0 ± 13.0 kg, respectively. The mean pre- and postoperative body mass indices (BMIs) were 25.3 and 25.9 kg/m^2^, respectively. We categorized patients based on the postoperative BMI according to underweight, normal, overweight, and obesity using the definition of obesity for Asians (18). The prevalence of obesity (≥25 kg/m^2^) was 62.5% (45/72), and that of obesity or overweight (≥23 kg/m^2^) was 87.5% (63/72). Only 12.5% (9/72) of the subjects were classified as normal weight (18.5–22.9 kg/m^2^), and none of the subjects were underweight (<18.5 kg/m^2^). Therefore, considering the high prevalence of HO in the present study, the power of this study is considered appropriate. During a median follow-up duration of 764 days (interquartile range 479–1724), the mean body weight change was 3.0 kg and mean BMI change was 1.3 kg/m^2^ (both *P*<0.01). The percentage changes in body weight and BMI were both 4.4%. The preoperative tumor volume was 4.6 (interquartile range 2.1–10.5) cm^3^.

**Table 1:** Baseline characteristics of study subjects.

Variables	*n* = 72
Age (years)	46.8 ± 14.8
Male, *n* (%)	41 (56.9)
Preoperative height (cm)	164.0 ± 9.6
Preoperative body weight (kg)	69.1 ± 13.0
Preoperative BMI (kg/m^2^)	25.3 (22.7–27.2)
Postoperative body weight (kg)	72.0 ± 13.0
Postoperative BMI (kg/m^2^)	25.9 (23.9–28.3)
Follow-up duration (days)	764 (479–1724)
Body weight change (kg)	3.0 (-0.9–6.2)
% body weight change (%)	4.4 (-1.1–10.1)
BMI change (kg)	1.3 (-0.3–2.4)
% BMI change (%)	4.4 (-1.1–10.0)
Previous history of surgery or irradiation for craniopharyngioma	16 (22.2)
Duration of steroid replacement (days)	719 (232–1456)
Daily steroid dose (mg/day)^a^	12.9 (10.9–15.3)
Cumulative steroid dose (mg)^a^	7295 (2962–12309)
Preoperative tumor volume (cm^3^)	4.6 (2.1–10.5)
Postoperative corrected hypothalamic volume^b^	0.042 ± 0.011
Preoperative visual disturbance, n (%)	52 (72.2)
Postoperative visual disturbance status	
Aggravation	4 (5.6)
No change	14 (19.4)
Improvement	50 (69.4)
Non-applicable	4 (5.6)
Preoperative hormone deficiency	
0	17 (24.6)
1	16 (22.2)
2	8 (11.1)
3	1 (1.4)
4	30 (41.7)
Postoperative hormone deficiency	
0	5 (6.9)
1	3 (4.2)
2	0 (0.0)
3	0 (0.0)
4	64 (88.9)
Postoperative diabetes insipidus, *n* (%)	62 (86.1)
Preoperative hypothalamic symptom, *n* (%)	25 (34.7)
Postoperative hypothalamic symptom, *n* (%)	9 (12.5)

Data are expressed as mean ± SD or median (interquartile range) or n (%). BMI, body mass index.

a Hydrocortisone-equivalent dose of steroid.

b Corrected hypothalamic volume = measured hypothalamic volume/temporal lobe volume.

With respect to HT volume, we used corrected postoperative HT volume from rater 1, and that was 0.042 ± 0.011 after adjusting for temporal lobe volume. Sixteen patients (22.2%) had a history of surgery and/or irradiation. Pre- and postoperative pituitary hormone deficiencies were observed in 76.3% and 93.0% of the patients, respectively. The median duration of steroid replacement therapy was 719 days (interquartile range 232–1456) with a median daily steroid (hydrocortisone) dose of 12.9 mg (interquartile range 10.9–15.3). Postoperative diabetes insipidus was reported in 62 patients (86.1%). Two patients required postoperative ventroperitoneal drainages due to uncontrolled hydrocephalus occurring before surgery.

### Correlation Analysis Between Corrected Postoperative HT Volume and Clinical Parameters of Study Subjects

We observed a significant negative association between the corrected postoperative HT volume and preoperative body weight (*r*=–0.25 [95% CI -0.45 to -0.04], *P*=0.04) (**Table 2A**). However, there were no associations between postoperative HT volume and postoperative body weight, body weight change, or percentage body weight change. We performed sensitivity analysis using BMI and changes in BMI. No associations were also observed regarding pre- and postoperative BMI, BMI change, or percentage BMI change (data not shown). The corrected postoperative HT volume showed a significant negative correlation with preoperative tumor volume (*r*=–0.26 [95% CI -0.40 to -0.15], *P*=0.03). Postoperative daily steroid dose (*r*=–0.32 [95% CI -0.49 to -0.06], *P=*0.01) and hypothalamic symptoms (*r*=–0.25 [95% CI -0.45 to -0.02], *P*=0.04) were also negatively associated with postoperative HT volume.

**Table 2:** Descriptive statistics and correlation matrix between corrected postoperative hypothalamic volume and clinical parameters.

	Corrected HT volume	Preop Bwt	Postop Bwt	Bwt change	% Bwt change	Preop tumor volume	Duration of steroid	Daily steroid dose *^a^*	Postop hormone deficiency	Postop hypothalamic symptoms
**(A) All patients (*n* = 72)**										
Corrected HT volume	1	–0.25^b^	-0.22	0.10	0.13	-0.26^b^	-0.01	-0.32^b^	-0.07	-0.25^b^
Preop Bwt	–0.25^b^	1	0.91	-0.35^b^	-0.43^b^	0.09	-0.11	0.05	0.13	0.13
Postop Bwt	–0.22	0.91^b^	1	0.07	-0.02	0.09	-0.13	0.13	0.13	0.15
Bwt change	0.10	-0.35^b^	0.07	1	0.98	-0.01	-0.03	0.17	-0.01	0.04
% Bwt change	0.13	-0.43^b^	-0.02	0.98^b^	1	-0.03	0.00	0.12	-0.00	0.01
Preop tumor volume	–0.26^b^	0.09	0.09	-0.01	-0.03	1	0.10	-0.00	0.13	0.12
Duration of steroid	–0.01	-1.11	-0.13	-0.03	0.01	0.10	1	-0.24^b^	0.27^b^	-0.11
Daily steroid dose *^a^*	–0.32^b^	0.05	0.13	0.17	0.12	0.00	-0.24^b^	1	-0.01	0.13
Postop hormone deficiency	–0.07	0.13	0.13	-0.01	-0.01	0.13	0.27^b^	-0.01	1	0.10
Postop hypothalamic Sx	–0.25^b^	0.13	0.15	0.04	0.01	0.12	-0.11	0.13	0.10	1
**(B) Patients who underwent primary surgery for CP (*n* = 56)**										
Corrected HT volume	1	–0.30^b^	-0.29^b^	0.07	0.10	-0.36^b^	-0.13	-0.18	-0.04	-0.13
Preop Bwt	–0.30^b^	1	0.90^b^	-0.34^b^	-0.43^b^	0.14	-0.14	0.20	0.13	0.29^b^
Postop Bwt	–0.29^b^	0.90^b^	1	0.10	-0.01	0.13	-0.14	0.33^b^	0.14	0.32^b^
Bwt change	0.07	-0.34^b^	0.10	1	0.98^b^	-0.04	0.03	0.25	0.01	0.02
% Bwt change	0.10	-0.43^b^	-0.00	0.98^b^	1	-0.06	0.07	0.16	0.01	-0.02
Preop tumor volume	–0.36^b^	0.14	0.13	-0.04	-0.06	1	0.14	0.11	0.16	0.11
Duration of steroid	–0.13	-0.14	-0.14	0.03	0.07	0.14	1	-0.14	0.30^b^	-0.01
Daily steroid dose *^a^*	–0.18	0.20	0.33^b^	0.25	0.16	0.11	-0.14	1	-0.12	0.11
Postop hormone deficiency	–0.04	0.13	0.14	0.01	0.13	0.16	0.30^b^	-0.12	1	0.10
Postop hypothalamic Sx	–0.13	0.29^b^	0.32^b^	0.02	-0.02	0.11	-0.01	0.11	0.1	1

HT, hypothalamus; Preop, preoperative; postop, postoperative; Bwt, body weight; Sx, symptom.

a Hydrocortisone-equivalent dose of steroid.

b Correlation is significant at the 0.05 level (two-tailed).

**Figure 3:**
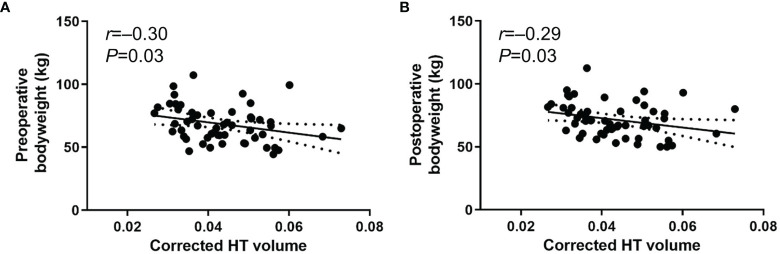
Scatter plots between body weight and corrected postoperative HT volume using method 2 from rater 1. **(A)** preoperative body weight **(B)** postoperative body weight in patients who underwent primary surgery for CP (*n*=56). Date are presented with 95% confidence interval.

To exclude the effect of pre-existing hypothalamic damage caused by prior surgery or radiation for CP, we performed a subgroup analysis in patients who underwent primary surgery for CP (*n*=56) (**Table 2B**). Pre- and postoperative body weights exhibited significant negative associations with the corrected postoperative HT volume (*r*=–0.30 [95% CI -0.53 to -0.03], *P*=0.03 and *r*=–0.29 [95% CI -0.53 to -0.02], *P*=0.03, respectively) (**Figures 3A, B**). Preoperative tumor volume also showed a negative association with postoperative HT volume (*r*=–0.36 [95% CI -0.53 to -0.25], *P*=0.01). However, we did not observe any significant associations between HT volume and body weight change and percentage body weight change. In addition, there were no associations between HT volume and pre- and postoperative BMI, BMI change, or percentage BMI change. Negative correlations between the corrected postoperative HT volume, daily steroid dose, and postoperative hypothalamic symptoms that were previously observed in all subjects were not present in this subgroup analysis.

Additionally, we performed adjusted multiple linear regression analysis to evaluate the independent association of HT volume with postoperative body weight after adjusting for preoperative body weight, duration of days between surgery and MRI/body weight measurement, preoperative tumor volume, and daily steroid dose. However, we did not find a significant association in the analysis in all patients (*n*=72; *β*=0.02*, P*=0.72) as well as in those who underwent primary surgery (*n*=56; *β*=-0.10*, P*=0.92).

We underwent sensitivity analysis using HT volume without temporal lobe volume normalization. In all patients (*n*=72), body weight change and percentage body weight change were positively associated with HT volume (*r*=0.36 [95% CI 0.18 to 0.52], *P*<0.01 and *r*=0.33 [95% CI 0.15 to 0.50], *P*<0.01). BMI change and percentage BMI change were also positively associated with HT volume (*r*=0.34 [95% CI 0.16 to 0.50], *P*<0.01 and *r*=0.33 [95% CI 0.15 to 0.50], *P*<0.01). Similar trends were observed in those who underwent primary surgery for CP (*n*=56). Body weight change and percentage body weight change were positively associated with HT volume (*r*=0.40 [95% CI 0.19 to 0.57], *P*<0.01 and *r*=0.35 [95% CI 0.14 to 0.54], *P*<0.01). BMI change and percentage BMI change were also associated with HT volume (*r*=0.39 [95% CI 0.19 to 0.56], *P*<0.01 and *r*=0.36 [95% CI 0.14 to 0.54], *P*<0.01). However, HT volume did not show significant associations with tumor volume, steroid replacement, or hypothalamic symptoms in both groups (data not shown). These findings support the need for temporal lobe volume normalization to accurately assess individual HT volume.

## Discussion

We developed a novel method of HT volume measurement using T2-weighted MR images to evaluate the extent of hypothalamic damage in adult patients with CP using an individually tailored segmentation method. In the present study, corrected postoperative HT volume was negatively associated with preoperative body weight, preoperative tumor volume, postoperative hypothalamic symptoms, and steroid daily dose in overall population. Of note, corrected postoperative HT volume was negatively associated with postoperative body weight as well as preoperative body weight and preoperative tumor volume in the primary surgery group, however, not with postoperative hypothalamic symptoms and steroid daily dose. We did not observe any significant correlations between the postoperative HT volume and postoperative weight gain parameters. In patients with CP, a few studies have assessed HT damage; however, the study subjects were limited to patients with childhood- or adolescent onset CP (12, 13). To the best of our knowledge, this is the first study to investigate HT volume measurements in adult-onset CP patients.

For HT volumetric assessments, previous studies mostly used established fixed landmarks and T1-weighted MR images. Several studies have attempted to evaluate HT volume manually (14, 19, 20) and semi-automatically (21–23). Further optimized methods have been performed by reviewing descriptions of hypothalamic anatomy (21, 24) and criteria from other segmentation protocols (24–26). For patients with CP having severe structural damage, established fixed landmarks create critical problems as they are often severely distorted and displaced outwardly owing to a large space in the third ventricle. To overcome this limitation, we developed a novel individualized segmentation method using new landmarks, including the thalamus. However, T1-weighted MR images has a critical limitation as the MR image of the thalamus may be obscured (27), which could lead to overestimation of HT measurement by incorporating the thalamus. Indeed, our results demonstrate this problem of volume overestimation (**Figure 1**). To overcome this limitation, we adopted T2-weighted MR images which can be used to clearly visualize the thalamus. With this approach, the upper margin of the HT was more accurate by avoiding the thalamus area. Furthermore, to precisely assess HT volume, we measured temporal lobe volume to correct the effect of whole brain size. Previous HT volumetric studies used specialized research setting for MR images with 0.5–1 mm slice thickness. This thin slice scan has better image quality and has been used for 3-dimentional reconstruction MR images. However, routine brain MRI for general clinical assessments usually does not use this thin thickness, including our hospital; MR images with 3 mm slice thickness are conventionally used in real-world clinical settings. Therefore, in the present study, we used MR images with 3 mm slice thickness. For wider usage of HT volume measurements, our new method may be more suitable for real-world practice in terms of accuracy and accessibility.

In the present study, the HT volume was measured using MRI after surgery and/or irradiation. Approximately 77.8% (56/72) of patients had no previous history of surgical resection or irradiation for CP. Hence, it can be concluded that the measured HT volume was a result of the hypothalamic damage caused by the tumor itself and the secondary damage related to the treatment for CP. Indeed, we observed a significant negative association between preoperative tumor volume and the HT volume, suggesting a mass effect of the tumor. Owing to the growth rate and location of CP, many patients presented with clinical manifestations of hypothalamic dysfunction at the time of diagnosis. Among the clinical manifestations, weight change, which is an important clinical sign of CP-related hypothalamic damage, can be overlooked. There is a possibility of undetected weight gain due to the mass effects of CP before diagnosis. However, it is not easy to reveal this issue in clinical practice owing to the unknown onset of CP and lack of reliable data on preoperative weight gain. In the present study, the HT volume showed a significant negative association with preoperative body weight, but not with parameters associated with postoperative weight change. These findings can be partially explained by the hypothesis that the effect of CP-induced hypothalamic injury on body weight is greater than that of surgical resection. Another explanation is the presence of bias resulting from subjects who previously underwent surgery or irradiation. Previous treatment-related hypothalamic damage and steroid replacement therapy might already have resulted in considerable weight gain prior to the subsequent surgery. To determine this, we performed correlation analysis specifically in CP subjects who underwent primary surgery. Interestingly, preoperative as well as postoperative body weight showed significant negative associations with the corresponding postoperative HT volumes. However, no significant associations were observed between body weight and the HT volume in subjects with a history of pretreatments for CP (data not shown).

This study has several limitations. First, the median follow-up duration of our study was 2 years. Although excessive weight gain usually occurs in the first year after surgical resection of patients with CP (28), HO is a long-term sequelae of CP; therefore, this interval may be short to fully represent the characteristics of HO. Second, we were unable to collect detailed information regarding eating behaviors or energy expenditure status of the study subjects owing to the retrospective nature of the study. Third, HT volume measurements were only performed using postoperative MR images. Therefore, we were unable to evaluate differences in pre- and postoperative HT volume or distinguish relative contributions of the tumor itself and surgical resection on hypothalamic damage. Fourth, we measured the total volume of HT to evaluate the degree of hypothalamic damage, but not the volume according to the detailed hypothalamic region. Considering the importance of affected hypothalamic nuclei for the occurrence of HO, a meticulous approach for the regional measurement of the HT volume seems to be ideal. However, we could not confirm the association between the HT volume and postoperative weight change and region-specific assessment of damaged HT. Fifth, the present study could not compare the present method to 1 mm slice thickness MRI images. Future studies with 1 mm slice thickness MRI images would provide direct comparisons regarding the performance of HT volume measurement using 1 mm or 3 mm slice thickness. Moreover, although the T2-weighted MR images showed superior results in HT volume measurement compared with the T1-weight MR images, it did not show robust associations with clinical parameters. Although the *P* value showed significant results in some parameters, the correlation coefficient was not as high as 0.3. There also may be false discoveries from the analysis due to multiple testing. Therefore, further validation studies are needed to better understand the clinical relevance of HT volume measurements assessing hypothalamic damage in patients with CP. Finally, only patients with adult-onset CP were included in the present study, hence, it is unclear whether this approach is beneficial for childhood-onset CP as well.

In conclusion, our study demonstrated a new method of HT volume measurement using T2-weighted MR images obtained from a conventional clinical setting using a 3 mm slice thickness for the MR images. In patients with CP, significant negative associations of postoperative HT volume with preoperative/postoperative body weight and tumor volume were observed, suggesting a relatively greater effect of tumor-induced hypothalamic injury than of surgically induced hypothalamic injury on body weight. Unlike previous HT volume measurement methods based on MR images with 1 mm slice thickness, the novel HT volume measurement method can be used in conventional MRI to provide a new, broader practical opportunity for the analysis of HO in the clinical setting.

## Data Availability Statement

The original contributions presented in the study are included in the article. Further inquiries can be directed to the corresponding author.

## Ethics Statement

The studies involving human participants were reviewed and approved by the Institutional Review Board of SNUH (IRB No. 1710-096-895). Written informed consent for participation was not required for this study in accordance with the national legislation and the institutional requirements.

## Author Contributions

HJC conceived and designed this research. ARH, ML, JHL, JHK, YHK, and HJC performed the data acquisition, analysis, and interpretation. ARH, ML, and HJC wrote the manuscript. ARH, ML, JHL, JHK, YHK, and HJC reviewed the full text. All the authors agreed with the final version of the manuscript.

## Conflict of Interest

The authors declare that the research was conducted in the absence of any commercial or financial relationships that could be construed as a potential conflict of interest.

## Publisher’s Note

All claims expressed in this article are solely those of the authors and do not necessarily represent those of their affiliated organizations, or those of the publisher, the editors and the reviewers. Any product that may be evaluated in this article, or claim that may be made by its manufacturer, is not guaranteed or endorsed by the publisher.
